# LUF7244, an allosteric modulator/activator of K_v_11.1 channels, counteracts dofetilide‐induced torsades de pointes arrhythmia in the chronic atrioventricular block dog model

**DOI:** 10.1111/bph.14798

**Published:** 2019-08-30

**Authors:** Muge Qile, Henriette D.M. Beekman, David J. Sprenkeler, Marien J.C. Houtman, Willem B. van Ham, Anna Stary‐Weinzinger, Stanislav Beyl, Steffen Hering, Dirk‐Jan van den Berg, Elizabeth C.M. de Lange, Laura H. Heitman, Ad P. IJzerman, Marc A. Vos, Marcel A.G. van der Heyden

**Affiliations:** ^1^ Department of Medical Physiology University Medical Centre Utrecht Utrecht The Netherlands; ^2^ Department of Pharmacology and Toxicology University of Vienna Vienna Austria; ^3^ Division of Systems Biomedicine and Pharmacology, Leiden Academic Centre for Drug Research Leiden University Leiden The Netherlands; ^4^ Division of Drug Discovery and Safety, Leiden Academic Centre for Drug Research Leiden University Leiden The Netherlands

## Abstract

**Background and Purpose:**

K_v_11.1 (hERG) channel blockade is an adverse effect of many drugs and lead compounds, associated with lethal cardiac arrhythmias. LUF7244 is a negative allosteric modulator/activator of K_v_11.1 channels that inhibits early afterdepolarizations in vitro. We tested LUF7244 for antiarrhythmic efficacy and potential proarrhythmia in a dog model.

**Experimental Approach:**

LUF7244 was tested in vitro for (a) increasing human I_Kv11.1_ and canine I_Kr_ and (b) decreasing dofetilide‐induced action potential lengthening and early afterdepolarizations in cardiomyocytes derived from human induced pluripotent stem cells and canine isolated ventricular cardiomyocytes. In vivo, LUF7244 was given intravenously to anaesthetized dogs in sinus rhythm or with chronic atrioventricular block.

**Key Results:**

LUF7244 (0.5–10 μM) concentration dependently increased I_Kv11.1_ by inhibiting inactivation. In vitro, LUF7244 (10 μM) had no effects on I_KIR2.1_, I_Nav1.5_, I_Ca‐L_, and I_Ks_, doubled I_Kr_, shortened human and canine action potential duration by approximately 50%, and inhibited dofetilide‐induced early afterdepolarizations. LUF7244 (2.5 mg·kg^−1^·15 min^−1^) in dogs with sinus rhythm was not proarrhythmic and shortened, non‐significantly, repolarization parameters (QTc: −6.8%). In dogs with chronic atrioventricular block, LUF7244 prevented dofetilide‐induced torsades de pointes arrhythmias in 5/7 animals without normalization of the QTc. Peak LUF7244 plasma levels were 1.75 ± 0.80 during sinus rhythm and 2.34 ± 1.57 μM after chronic atrioventricular block.

**Conclusions and Implications:**

LUF7244 counteracted dofetilide‐induced early afterdepolarizations in vitro and torsades de pointes in vivo. Allosteric modulators/activators of K_v_11.1 channels might neutralize adverse cardiac effects of existing drugs and newly developed compounds that display QTc lengthening.

**Abbreviations**
APD: action potential duration
CAVB: chronic atrioventricular block
CMCs: cardiomyocytes
EAD: early afterdepolarization
hERG: human ether‐a‐go‐go‐related gene
hiPSC: human induced pluripotent stem cell
I_Kr_: rapid component of the delayed rectifier potassium outward current
K_v_11.1: voltage gated potassium channel carrying I_Kr_
LV: left ventricle
MAPD: monophasic action potential duration
MD: molecular dynamics
QTc: heart rate‐corrected ECG QT interval
RV: right ventricle
SR: sinus rhythm
STV: short‐term variability of repolarization duration
TdP: torsades de pointes

**What is already known**

- Drug‐induced torsades de pointes has limited the use of several otherwise effective drugs.
- Allosteric modulators can lead to a change in receptor conformation.

**What does this study adds**

- LUF7244 reduced 71% of dofetilide‐induced torsades de pointes in dogs with chronic atrioventricular block.

**What is the clinical significance**

- LUF7244 may rescue drugs withdrawn due to I_Kr_ blocking side‐effects, via combination therapy.

## 1 INTRODUCTION

Drug‐induced torsades de pointes (TdP) can degenerate into ventricular fibrillation and sudden cardiac death. This toxicity generated strict regulations that limited the use of several existing, otherwise efficient drugs, along with difficulties in the development of new drugs (Salvi, Karnad, Panicker, & Kothari, 2010). The structural characteristics of the K_v_ 11.1 (human ether‐a‐go‐go‐related gene [hERG]) channel, responsible for the delayed rectifier K^+^ current I_Kr_, make it a common target for a variety of drugs which frequently results in unintended cardiac side effects, such as the TdP arrhythmias (Perry, Sanguinetti, & Mitcheson, 2010; Sanguinetti & Tristani‐Firouzi, 2006). Blockade of the I_Kr_ prolongs action potential duration (APD), which is manifested as a lengthening of the QT interval on the ECG. This effect is considered as a surrogate marker of cardiotoxicity and for this reason incorporated in regulatory agency guidelines (i.e., ICH‐S7B, ICH‐E14, and EMEA). Many drugs, although effective for their intended application, have been withdrawn from the market, such as astemizole and cisapride or are restricted in their use, such as terfenadine and haloperidol, due to observed QT prolongation and/or the induction of TdP (Darpö, 2001). Furthermore, the current, stringent preclinical guidelines have stopped the development of promising drug candidates, because they block I_Kr_ and/or lengthen QT interval. Therefore, initiatives to develop effective agents to counteract those side effects of (preclinical) drugs are ongoing (Grunnet, Hansen, & Olesen, 2008), and their efficacy and safety in vivo need to be established.

Allosteric modulators are a class of ligands that bind to an allosteric site and thereby modulate the binding of an orthosteric ligand on the receptor. Compared with a normal, competitively binding agent, allosteric modulators tend to display higher subtype selectivity and a ceiling effect (Conn, Christopoulos, & Lindsley, 2009). Negative allosteric modulators for K_v_11.1 channels would reduce affinity of the ion channel for its inhibitors and counteract drug‐induced blockade. Recently, LUF7244, one out of a series of novel negative allosteric modulators of K_v_11.1 channels, was synthetized, and some of its functional properties were analysed (Yu, Klaasse, Heitman, & IJzerman, 2014). LUF7244 might be able to prevent cardiac proarrhythmias due to I_Kr_ blockade in vivo because it (a) decreased the affinity of the K_v_11.1 channel blockers cisapride, astemizole, dofetilide, and sertindole for binding to these channels and (b) induced APD shortening in neonatal rat ventricular myocytes and inhibited early afterdepolarization (EAD) formation following astemizole exposure (Yu et al., 2016).

I_Kr_ has an important role in cardiac repolarization in the dog. Specific blockade of the current carried by K_v_11.1 channels results in a marked increase in QTc (see Opstal, Leunissen, Wellens, & Vos, 2001; Schneider, Hauser, Andreas, Linz, & Jahnel, 2005). In the chronic atrioventricular block (CAVB) dog model, repolarization is further compromised (Schoenmakers et al., 2003) resulting in high propensity for ventricular arrhythmias upon pharmacological blockade of I_Kr_ (Oros, Beekman, & Vos, 2008). These characteristics make the CAVB dog model appropriate for defining potential antiarrhythmic properties of newly developed pharmacological agents (Bossu et al., 2018), which might apply to negative allosteric modulators of K_v_11.1 channels as well.

The research described here aimed (a) to use relevant cellular models (i.e., cardiomyocytes derived from human induced pluripotent stem cells (hiPSC) and ventricular cardiomyocytes, isolated from dogs) in which K_v_11.1 channels make an important contribution to APD and, therewith, study the effects of LUF7244 application on human‐(like) cellular electrophysiology and (b) to determine antiarrhythmic capacities of LUF7244 administration in CAVB dogs against dofetilide‐induced TdP arrhythmias.

## 2 METHODS

### 2.1 In vitro studies

#### 2.1.1 Cell culture

For determining the effects of LUF7244 on I_Kv11.1_, I_KIR2.1_, and I_Nav1.5_, stable HEK293 (CLS Cat# 300192/p777_HEK293, RRID:CVCL_0045) cell lines were used instead of isolated canine ventricular cardiomyocytes. The HEK293 cells were used here because, for the latter, determination of the individual currents requires specific pharmacological isolation of the current for which potential pharmacological interactions with LUF7244 are unknown. The HEK‐hERG cell line (Zhou, Gong, Epstein, & January, 1998) was derived from HEK‐293T cells and stably expresses human K_v_11.1 protein. The HEK‐KWGF cell line stably expresses C‐terminal GFP‐tagged murine K_IR_2.1 (De Boer et al., 2006). The HEK‐Na_v_1.5 cell line (a kind gift of Dr. H. Abriel, Bern University, Switzerland) stably expresses human Na_v_1.5. HEK‐hERG cells, KWGF cells, and HEK‐Na_v_1.5 were cultured in DMEM with 10% fetal calf serum, 2‐mM l‐glutamine, 50 U·ml^−1^ penicillin, and 50 mg·ml^−1^ streptomycin and passaged twice a week. During experiments, the amount of DMSO added to the cells was below 0.1%.

#### 2.1.2 Optical action potential measurements in hiPSC‐CMCs

We used commercial Cor.4U® human‐induced pluripotent stem cell‐derived cardiomyocytes (hiPSC‐CMCs; Ncardia, Cologne, Germany) as described before (Baburin et al., 2018). Cells were transiently loaded with the voltage‐sensitive dye FluoVolt, and the multiwell plate was placed in an environmentally controlled stage incubator (37°C, 5% CO_2_, water‐saturated air atmosphere; Okolab Inc, Burlingame, CA, USA). The FluoVolt fluorescence signals were recorded from 0.2 × 0.2‐mm areas using a 40× (NA 0.6) objective lens at excitation wavelength 470 ± 10 nm provided by a LED, and emitted light was collected by photomultiplier at 510–560. LED, photomultiplier, associated power supplies, and amplifiers were supplied by Cairn Research Ltd (Kent, UK). Fluorescence signals were digitized at 10 kHz; 20‐s recordings of spontaneous APs were taken in single drug and competition protocols and repeated five times. pClamp (RRID:SCR_011323) software package v.10.0 (Molecular Devices, Inc., Sunnyvale, CA, USA) was used for offline analysis of APD at 90% of repolarization. To investigate possible time‐dependent effect of vehicle on AP parameters, we compared APs in control and in the presence of vehicle 5, 15, and 30 min after vehicle application. All estimated AP parameters (APD_30_, APD_50_, and APD_90_) in control and at the presence of vehicle were statistically not significantly different at any measured time point (data not shown).

#### 2.1.3 Patch clamp

Current was recorded with an Axon‐patch 200B amplifier (Molecular Devices, CA, USA) and analysed with ClampFit 10.2 software (Molecular Devices). I_Kv11.1_, I_KIR2.1_, I_Nav1.5_, and I_Ca‐L_ were measured under room temperature (22°C). Action potential and I_K_ were measured at 37°C.

##### I_Kv11.1_ recording

HEK‐hERG cells were grown on Ø12‐mm cover slips coated with 0.1% gelatin. Cells were perfused with Tyrode's solution (mM): 140 NaCl, 4 KCl, 10 HEPES, 2 CaCl_2_, 1 MgCl_2_ (pH 7.4, NaOH). The intracellular (pipette) solution contained (mM): 10 EGTA, 110 KCl, 10 HEPES, 4 K_2_‐ATP, 5.17 CaCl_2_, 1.42 MgCl_2_ (pH 7.2, KOH). K_v_11.1 current was measured with whole cell voltage clamp and its pulse protocol ranging from −80 to +60 mV for 4,000 ms followed by a 5,000‐ms deactivation pulse at −50 mV. The interpulse interval was 10 s (0.1 Hz). Cell capacitance and series resistance were calculated in each cell.

##### I_KIR2.1_ recording

KWGF cells were cultured on coated cover slips. I_KIR2.1_ measurements were performed in bath solution containing (mM): 140 NaCl, 5.4 KCl, 1 CaCl_2_, 1 MgCl_2_, 17.5 NaHCO_3_, 15 HEPES, 6 glucose (pH 7.4, NaOH). Pipette solution contained (mM): 125 K‐gluconate, 10 KCl, 5 HEPES, 5 EGTA, 0.6 CaCl_2_, 2 MgCl_2_, 4 Na_2_ATP (pH 7.2, NaOH). I_KIR2.1_ was measured by 1‐s voltage steps from −120 to +30 mV with 10‐mV increments, from a holding potential of −40 mV. The interpulse interval was 3 s (0.33 Hz). Signals were low pass filtered at 2 kHz.

##### I_Nav1.5_ recording

I_Nav1.5_ was recorded at 20°C from HEK‐Na_v_1.5 cells. The extracellular solution contained (mM): 140 NaCl, 5 CsCl, 1.8 MgCl_2_, 5 glucose, 5 HEPES, and 0.002 nifedipine (pH 7.3, CsOH). Pipette solution contained (mM): 5 NaCl, 133 CsCl, 2 MgATP, 20 tetraethylammonium chloride, 10 EGTA, 5 HEPES (pH 7.3, CsOH). Sodium currents were measured by 20‐ms step pulses ranging from −80 to +40 mV, from a holding potential of −120 mV and an interpulse interval of 4 s (0.25 Hz).

##### Action potential recording

Only isolated CMCs that were rod‐shaped with clear striation and sharp edges and that showed no spontaneous activity were used. Action potentials were triggered in whole cell current clamp mode with 2‐ms current injections at 0.5 Hz and recorded. All measurements were performed at 37°C using a temperature‐controlled perfusion chamber (Cell Microcontrols). Tyrode's solution was used as bath solution containing (mM): 137 NaCl, 5.4 KCl, 1.8 CaCl_2_, 11.8 HEPES, 10 glucose, pH 7.4 adjusted with NaOH. Pipette solution contained (mM) 10 NaCl, 130 KCl, 10 HEPES, 5 MgCl_2_, pH 7.2 adjusted with KOH. For prevention experiments, four different concentrations of LUF7244 were administered to CMCs via an additional rapid solution exchange system (ALA Scientific Instruments, Long Island, NY, USA). For suppression experiments, four concentrations of LUF7244 were perfused subsequent to EADs occurrence after 1‐μM dofetilide perfusion. Incidence of EADs was calculated for each group. All APD (measured at 80% of repolarization) and short‐term variability of repolarization duration (STV) were analysed with Matlab (Mathworks, MA, USA).

##### I_K_ recording

For I_K_ measurements on CMCs, bath solution contained (mM): 4 KCl, 145 NaCl, 1.8 CaCl_2_, 1 MgCl_2_, 10 HEPES, 11 glucose, 0.005 nifedipine (pH 7.4, NaOH) and pipette solution contained (mM): 125 K‐aspartate, 1 MgCl_2_, 5 MgATP, 5 HEPES, 10 EGTA, 20 KCl (pH 7.2, KOH) was used. I_K_ was recorded using step pulses ranging between −20 and +60 mV, from a holding potential of −80 mV.

##### I_Ca‐L_ recording

L‐type calcium currents were measured in bath solution containing (mM): 140 NMDG, 4 KCl, 1 CaCl_2_, 1 MgCl_2_, 6 glucose, 17.5 NaHCO_3_, 15 HEPES (pH 7.4, HCl). The pipette solution contained (mM): 120 CsCl, 10 tetraethylammonium chloride, 1 CaCl_2_, 3 MgCl_2_, 2 Na_2_ATP, 10 EGTA, 5 HEPES (pH 7.2, CsOH). I_Ca‐L_ was recorded by applying 500‐ms pulses ranging from −60 to +40 mV with 10‐mV increments, from a holding potential of −80 mV.

#### 2.1.4 Molecular modelling

Molecular docking was carried out using the K_v_11.1 cryo‐EM structure (PDB code: 5va1) and GOLD (RRID:SCR_000188) v.5.6.2 (Jones, Willett, Glen, Leach, & Taylor, 1997). Given the similarity of LUF7244 to the known K_v_11.1 channel activator ICA‐105574, we assumed that they might share overlapping/related binding sites. Thus, residue F557 of one of the four subunits was set as binding site, with a binding radius of 10 Å. Residues F557, F619, T623, Y652, and F656 within the radius were set to flexible. For the two scoring functions used, ChemPLP and Goldscore, 100 runs were done each, with 125.000 Gold algorithm operations. The top 15 scoring poses of both functions were inspected and reviewed on their proposed interactions. Visualization of results was done using PyMol (RRID:SCR_000305) 1.7.2 (Schrödinger, 2017).

#### 2.1.5 Molecular dynamics simulations

Building of the molecular dynamics (MD) system as well as ligand parametrization was done using CHARMM‐GUI (Jo, Kim, Iyer, & Im, 2008). The K_v_11.1 hERG structure was embedded in a POPC bilayer and solvated with TIP3P water, and ions were added to create a KCl concentration of 0.15 M. K^+^ ions in the selectivity filter were placed at sites S0, S2, and S4, with water molecules at sites S1 and S3. Energy minimization, 20‐ns equilibration and production runs were performed using GROMACS (RRID:SCR_014565) v.5.1.2 (Abraham, Hess, van der Spoel, & Lindahl, 2015). The coordinates of the docking pose of LUF7244 were implemented in the system. Production runs were performed for 50 ns with the charmm36 forcefield (Vanommeslaeghe et al., 2010). Electrostatics were modelled using Particle Mesh Ewald (Darden, York, & Pedersen, 1993), and LINCS was used to constrain H‐bonds (Hess, Bekker, Berendsen, & Fraaije, 1997). Temperature was maintained at 310 K using V‐rescale (Bussi, Donadio, & Parrinello, 2007), and semi‐isotropic pressure coupling was done using the Parrinello‐Rahman barostat (Parrinello & Rahman, 1981). MD trajectories were analysed using VMD v.1.9.2 (Humphrey, Dalke, & Schulten, 1996) and GROMACS.

### 2.2 In vivo studies

#### 2.2.1 Animals

All animal care and experimental procedures were approved by the Committee for Experiments on Animals of Utrecht University and conformed to the Directive 2010/63/EU of the European Parliament. Animal studies are reported in compliance with the ARRIVE guidelines (Kilkenny, Browne, Cuthill, Emerson, & Altman, 2010) and with the recommendations made by the *British Journal of Pharmacology*. The current study has no implications for replacement, refinement, or reduction.

#### 2.2.2 Animal handling

Dogs were housed in pairs in kennels and provided with wooden bedding material. Dogs had access to water ab libitum and received dog food pellets twice a day. Playing toys were provided as enrichment. Dogs were allowed to play outside in groups once a day. Welfare assessment was checked every day, and the body weight was monitored once a week. Animals were fasted for the day before surgery (from 4:00 p.m.).

**Figure 1:**
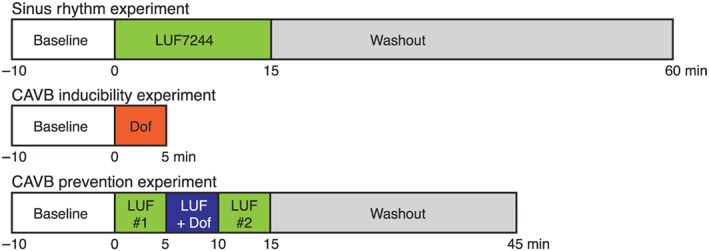
Experimental set‐up for in vivo studies

A total of 10 adult mongrel dogs (Marshall, New York, USA; four females and six males; body weight 24 ± 4 kg; 13 ± 3 months old) were included for serial experiments with at least 2 weeks in between as a recovery interval (Figure 1). Sex‐associated differences in QTc have not been documented in this species (Salama & Bett, 2014), and as per our unpublished observations, sex has no influence on the dofetilide inducibility of arrhythmias. Premedication included atropine (i.m. 0.02 mg·kg^−1^), methadone (i.m. 0.5 mg·kg^−1^), and acepromazine (i.m. 0.5 mg·kg^−1^). To limit risk of infection, antibiotic was given (ampicillin 1,000 mg, before and after the operation, intravenously and intramuscularly, respectively). Analgesia was provided by buprenorphine (Temgesic, 0.3 mg i.m.) after operation. General anaesthesia was induced by pentobarbital sodium (Nembutal, 25 mg·kg^−1^, i.v.) and maintained by 1.5% isoflurane in a mixture of O_2_ and N_2_O (1:2). Atrioventricular block was induced by radiofrequency ablation as described before (Oros et al., 2008).

#### 2.2.3 Drug dosing

The hERG activator ICA‐105574 shows structural similarities with LUF7244 and has been analysed in anaesthetized dogs at 1 and 10 mg·kg^−1^·10 min^−1^, previously (Asayama et al., 2013). ICA‐105574 caused QTc shortening only at the highest concentration. Based on these results, we decided for conservative application and infused LUF7244 at 2.5 mg·kg^−1^·15 min^−1^ in the in vivo experiments. In total, 10‐ml LUF7244 (2.5 mg·kg^−1^, i.v.) has been infused at a 40 ml·hr^−1^ infusion rate. LUF7244 and dofetilide were infused by two different front limb (intravenously) and two separate syringes in an infusion dock.

#### 2.2.4 Dogs with sinus rhythm

Under general anaesthesia, after monophasic action potential (MAP) catheters were introduced into the left ventricle (LV) and right ventricle (RV), a 10‐min baseline recording was started followed by a 15‐min LUF7244 infusion (2.5 mg·kg^−1^). The infusion protocol was followed by 30‐min washout (Figure 1, top panel). During the entire loading and washout procedure, ECG parameters and LV/RV MAP were recorded. Blood samples were collected every 5 min to determine the plasma level of LUF7244.

#### 2.2.5 Dogs with CAVB

Ventricular remodelling following CAVB was allowed for at least 2 weeks under idioventricular rhythm (Oros et al., 2008) before serial experiments were conducted (Figure 1).

#### 2.2.6 Inducibility experiment

Dofetilide infusion (0.025 mg·kg^−1^·5 min^−1^) was stopped either after 5‐min of infusion or with the first occurrence of TdP (Figure 1, middle panel). Dogs that showed at least three TdPs within 10 min after the start of dofetilide infusion were considered as inducible. Dogs were defibrillated when severe, not self‐terminating arrhythmia events occurred. ECG and LV/RV MAP signals were recorded during the whole procedure.

#### 2.2.7 Prevention experiment

Only inducible dogs were selected to undergo the subsequent prevention experiment. After 10‐min baseline recording, a 15‐min infusion of LUF7244 (2.5 mg·kg^−1^) was started, during which time dofetilide (0.025 mg·kg^−1^) was infused between *t* = 5 min and *t* = 10 min (Figure 1, bottom panel). The ECG and LV/RV MAP signals were recorded. Blood samples were collected during the infusion periods (*t* = 0/15/30/45 min).

#### 2.2.8 Data analysis

ECG parameters from lead II (RR, PQ, QRS, and QT) were measured manually with EPTracer (Cardiotek, Maastricht, the Netherlands) and were quantified every 5 min from start of baseline recording. Electrophysiological parameters were the average from five consecutive beats (except RV/LV STV were based on 30 consecutive beats). ECG intervals and MAP signals were recorded with a sampling rate of 1 kHz. QT interval was corrected by Van de Water's formula QTc = QT − 0.087 * (RR − 1,000). LV and RV MAPD80 were analysed semi‐automatically with the custom‐made software AutoMAPD (Matlab (RRID:SCR_001622), Mathworks, MN, USA). Beat‐to‐beat variability of repolarization was quantified as STV. It (based on 30 consecutive beats) was calculated with the formula STV =  ∑  ∣ *D*_*n*+1_ − *D*_*n*_ ∣ /(30 *  √ 2) in which *D* represents APD. Arrhythmia score (Stams et al., 2016) was used to quantify arrhythmia severity. The arrhythmia score is the average of three most severe arrhythmia events during 10 min. One regular beat scored as 1 point, and single ectopic beats were counted as 2 points. Multiple ectopic beats were scored 3–50 points. One, two, or more defibrillations scored as 50, 75, and 100. In the absence of arrhythmic events in all animals within one group, arrhythmia score is 1 with *SD* of 0.

Animals were not randomized in the present study. Each dog served as its own control under sinus rhythm (SR) and CAVB conditions. Addition of another group of animals (continuously receiving saline/vehicle) would not be justified ethically (3Rs) or scientifically. In this study, operators and data analysts were not blinded. In SR dog experiments, all dogs received LUF7244 consecutively for 15 min. In CAVB dog inducibility experiments, dofetilide was administered in all dogs and resulted in at least three TdP arrhythmias of 5 or more beats in all dogs. TdP arrhythmias are immediately evident on the surface ECG running during the experiments. In CAVB dog prevention experiments, LUF7244 and dofetilide were administered in all dogs. Successful suppression of TdP upon LUF7244 infusion is evident during the experiment (ECG monitor) and from the recorded ECG. Therefore, blinding is not applicable.

#### 2.2.9 Isolation of CMCs

Following the final in vivo experiment, heparin (10,000 I.U., i.v.) was administered, right‐sided thoracotomy was performed, and the heart was excised. For the current study, ventricular CMCs were enzymically isolated from 44 dogs (Table S2), either from dogs in the currently described protocol or from dogs present in other approved study protocols from our institute, as described before (Varkevisser et al., 2012). The excised heart was washed with Ca^2+^ free buffer (mM): 130 NaCl, 5.4 KCl, 1.2 KH_2_PO_4_, 1.2 MgSO_4_, 6 HEPES, 20 glucose (pH 7.2, NaOH). The left coronary artery was ligated into the Langendorff system and perfused with warm (37°C) Ca^2+^ free buffer solution for 10 min and then perfused for 25–35 min with the enzymic solution containing (420‐mg collagenase A; Roche, the Netherlands) and 32‐mg protease (Sigma‐Aldrich, the Netherlands) in 2.5% trypsin 400 μl, in 400‐ml buffer solution. Finally, 0.2‐mM Ca^2+^ buffer solution was perfused for 10 min. Ventricular CMCs were harvested from midmyocardial tissue of ventricular free wall and filtered. The freshly obtained CMCs were stored in Ca^2+^ buffer solution at room temperature until use.

#### 2.2.10 Plasma concentration analysis

The analysis of LUF7244 was performed with UHPLC and UV detection. Separation was performed on a C‐18 UHPLC Kinetex EVO column (100 × 2.1 mm, 2.6 μm) from Phenomenex (Utrecht, the Netherlands). The composition of the mobile phase was 34% acetonitrile with 0.2% formic acid (v/v) which was used at a flow of 0.8 ml·min^−1^ and a temperature of 30°C. Sample preparation was performed by mixing 50 μl of plasma with 50 μl of mobile phase. After vortexing briefly with 1,000 μl of acetonitrile, the samples were centrifuged at 20,000× *g* during 10 min. The supernatants were evaporated in a Centrivap vacuum centrifuge from Labconco (Kansas City, Missouri). The samples were reconstituted in 50 μl of mobile phase (34% acetonitrile, 0.2% formic acid) by vortexing briefly at high speed. Subsequently, after centrifuging at 20,000× *g* for 10 min, 45 μl of the supernatant of each sample was transferred to a glass microinsert (31 × 6 mm) from BGB science (Harderwijk, the Netherlands). A volume of 10 μl was injected onto the column using a Nexera‐I HPLC system from Shimadzu ('s‐Hertogenbosch, the Netherlands).

Calibration was performed by a calibration curve in mobile phase (34% acetonitrile with 0.2% formic acid [v/v]). The concentrations used for calibration were 10, 20, 50, 100, 200, 500, and 1,000 ng·ml^−1^. To that end, LUF7244 was dissolved in methanol with 0.05% formic acid (v/v) and diluted in order to prepare the calibration solutions. Blank dog plasma aliquots were mixed with these calibration solutions and further processed as samples. Quality controls were prepared at the concentration levels of a low, medium, and high concentration (50, 200, and 1,000 ng·ml^−1^). The quality controls were stored in the −80°C freezer until used for quality control along with the actual sample concentration measurements.

### 2.3 Data and statistical analysis

The data and statistical analysis comply with the recommendations on experimental design and analysis on pharmacology (Curtis et al., 2018). Data are represented as mean ± *SD* or mean ± *SEM*. Two‐way ANOVA and Bonferroni post hoc test were used for APD_90_ results from hiPSC. Post hoc tests were carried out only if *F* was significant and there was no variance in homogeneity. A non‐parametric paired *t* test was used for group comparisons and arrhythmia score in the in vivo study. All statistical analyses were performed by Prism 6 (GraphPad, CA, USA; GraphPad Prism, RRID:SCR_002798). *P* values <.05 were considered as statistically significant.

### 2.4 Materials

LUF7244 was synthesized according to a previously published procedure (Yu et al., 2014). For cellular experiments, LUF7244 was dissolved in DMSO to obtain a stock solution of 100 mM that was filter sterilized (0.22 μm) and stored at −20°C until use. The amount of DMSO added to the cells was below 0.1%. For use in animals, LUF7244 was dissolved in DMSO and polyethylene glycol 400 (1:1, v/v) solution to final concentration and was filter sterilized (0.45 μm). The final solution is 10 ml in total (5‐ml DMSO and 5‐ml polyethylene glycol 400). The I_Ks_ blocker HMR1556 (Sanofi Aventis, Gouda, the Netherlands) was dissolved in DMSO at 10 mM as a stock solution and stored at −20°C until use.

For cellular experiments, dofetilide (Sigma‐Aldrich, Zwijndrecht, the Netherlands) was dissolved in DMSO at 10 mM as stock solution and stored at −20°C. For in vivo experiments, dofetilide was dissolved in 100‐μl HCl (0.1 M) and then diluted into saline (19–29 ml, depends on body weight) to the final concentration. Solutions for the in vivo experiments were freshly prepared before using. All the other chemicals were of analytical grade and obtained from standard commercial sources.

For the plasma sample analysis of LUF7244, methanol, acetonitrile, and formic acid were used in LC–MS grade and purchased from Biosolve (Valkenswaard, the Netherlands). Nanopure lab water was derived from an ELGA Purelab Flex water purification system from Veolia Nederland (Nieuwegein, the Netherlands).

### 2.5 Nomenclature of targets and ligands

Key protein targets and ligands in this article are hyperlinked to corresponding entries in http://www.guidetopharmacology.org, the common portal for data from the IUPHAR/BPS Guide to PHARMACOLOGY (Harding et al., 2018), and are permanently archived in the Concise Guide to PHARMACOLOGY 2017/18 (Alexander et al., 2017).

## 3 RESULTS

### 3.1 LUF7244 is predicted to bind K_v_11.1 channels and increased I_Kv11.1_ in HEK293 cells

**Figure 2:**
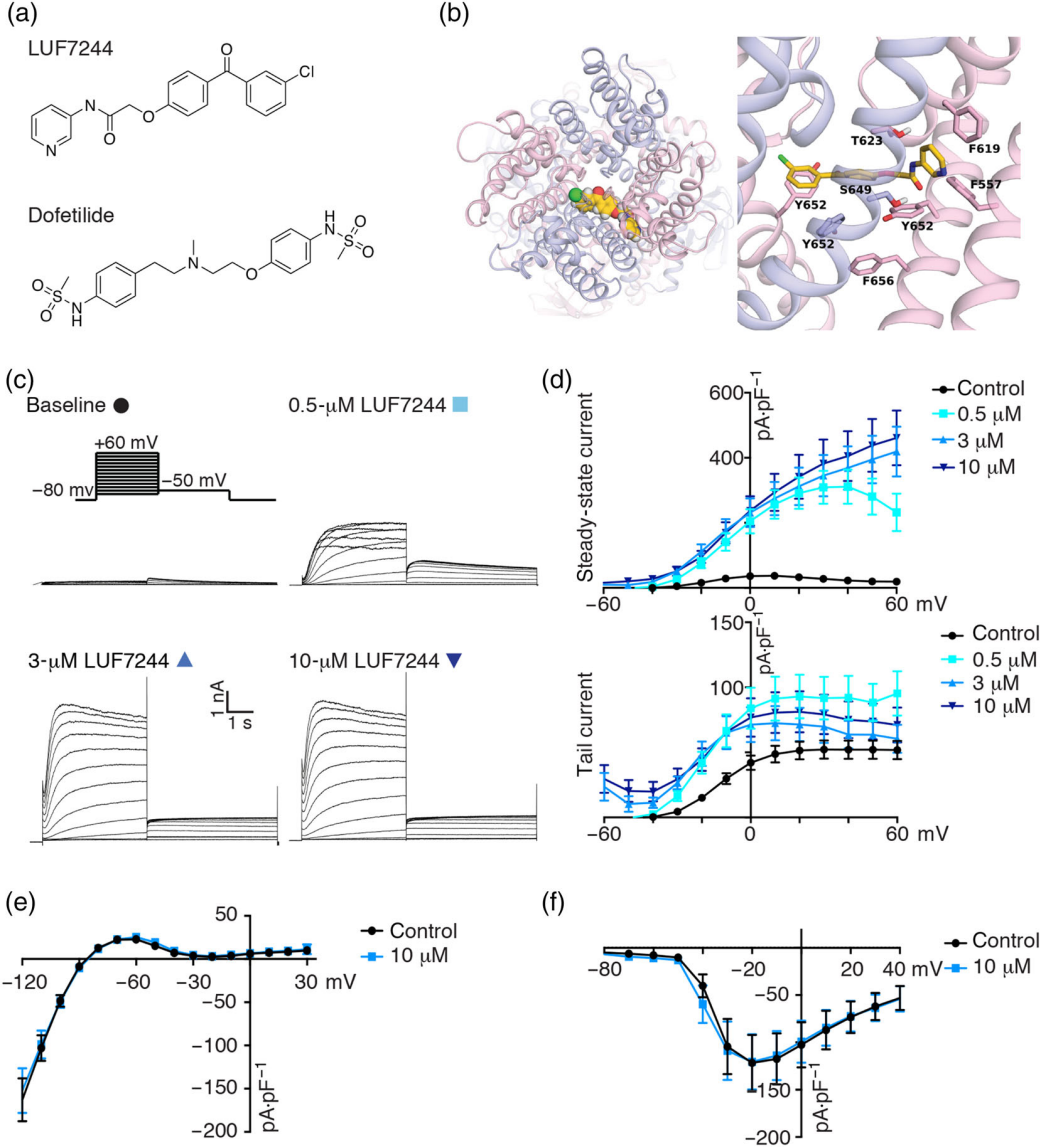
LUF7244 increases steady‐state I_Kv11.1_ in HEK‐hERG cells. (a) Chemical structure of LUF7244 and dofetilide. (b) Docking pose of LUF7244, after 50 ns of molecular dynamics simulation to the cryo‐EM structure of hERG. (c) Representative traces of K_v_11.1 current with or without infusion of LUF7244 (0.5, 3, and 10 μM). Stimulation protocol is shown on top panel. (d) I‐V relationship curve of I_Kv11.1_ under baseline conditions and in the presence of LUF7244 (0.5, 3, and 10 μM; n = 14, 13, and 12 cells). (e) I‐V curve of I_KIR2.1_ currents obtained from KWGF cells under baseline conditions and in the presence of 10‐μM LUF7244 (n = 9 cells). (f) I‐V relationship curve of I_Nav1.5_ measured in HEK‐Na_v_1.5 cells under baseline conditions and 10‐μM LUF7244 treatment (n = 10 cells). Raw current data were normalized by each cell's membrane capacitance for current density, for comparisons. Values are shown as mean ± SEM

Docking and subsequent MD studies suggested LUF7244 (Figure 2b) binds between the K_v_11.1 pore helices of two adjacent subunits, thereby stabilizing the channel in the conductive state (Figure 2b). In the predicted binding mode, the compound favourably interacts with aromatic residues F557 (S5), F619 (P‐helix), and Y652 residues (S6) from neighbouring subunits. Further, close contacts to selectivity filter residue T623 and S649 (S6) are predicted. The functional effects of LUF7244 on human I_Kv11.1_ were tested on the HEK‐hERG cell line by use of whole‐cell patch clamp. Three different concentrations of LUF7244 (0.5, 3, and 10 μM) were applied to cells resulting in a concentration‐dependent increase of the steady‐state current at the end of the depolarizing current (Figure 2c,d). Moreover, LUF7244 affected rectification and altered tail current kinetics upon the deactivation pulse (Figure 2c,d); 10‐μM LUF7244 had no effect on I_KIR2.1_ or I_Nav1.5_ (Figure 2e,f).

### 3.2 LUF7244 reduced APD and dofetilide‐induced prolongation in hiPSC‐CMCs

**Figure 3:**
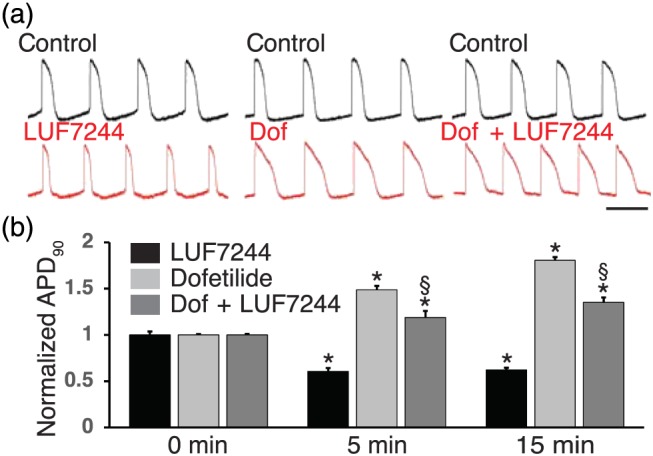
LUF7244 shortens APD and suppresses APD prolongation in hiPS‐CMCs. (a) AP recordings in control and after 5‐min incubation with the indicated drug or drug combinations (red) at 10 μM LUF7244 and 30 nM dofetilide. Horizontal bar indicates 1 s. (b) The bar graphs represent changes in normalized APD_90_ observed with each drug, or drug combination tested (n = 5). Values are shown as mean ± SEM. *P < .05, significantly different from the same drug(s) at 0 min. ^§^ P < .05, dofetilide + LUF7244 significantly different from dofetilide

We characterized the effects of LUF7244 on action potentials of human origin in hiPS‐CMCs using the voltage‐sensitive fluorescent dye FluoVolt. LUF7244 (10 μM) significantly shortened the APD at 90% of the repolarization phase (APD_90_; Figure 3a, left, and 3b). In hiPS‐CMCs challenged with 30‐nM dofetilide, APD was profoundly increased after 5 and 15 min (Figure 3a, middle, and 3b). LUF7244 co‐application decreased dofetilide‐induced APD prolongation, although values did not return to baseline (Figure 3a, right, and 3b). Vehicle time controls did not affect AP parameters.

### 3.3 LUF7244 shortened APD and reduced dofetilide‐induced EAD in isolated canine CMCs

**Figure 4:**
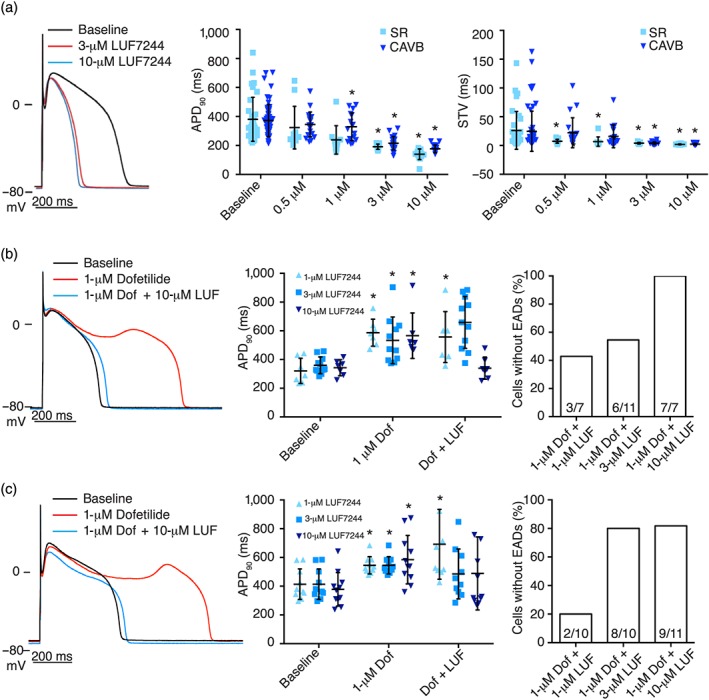
LUF7244 shortens APD and reduces dofetilide‐induced EAD in isolated canine cardiomyocytes. (a) Representative examples of LUF7244 induced shortening of APD in cardiomyocytes (left panel). Summarized results of APD_90_ (middle panel) and STV (right panel) after LUF7244 application to SR and CAVB cardiomyocytes . In SR cardiomyocytes , n = 11, 10, 13, and 14 cells were treated with 0.5, 1, 3, and 10 μM of LUF7244, respectively. In CAVB cardiomyocytes , n = 20, 16, 22, and 17. (b) LUF7244 dose dependently suppressed dofetilide‐induced EAD in SR cardiomyocytes (left panel). Summarized results of APD_90_ (middle panel) and incidence of EADs after dofetilide infusion in SR CMCs (right panel). Data are represented as number of cells without EADs/total number of cells showing EADs upon dofetilide. n = 7, 11, and 7 cells for 1, 3, and 10 μM of LUF7244, respectively. (c) One, 3, and 10 μM of LUF7244 suppressed dofetilide‐induced APD prolongation and EADs in CAVB cardiomyocytes . Summarized results of APD_90_ (middle panel) and incidence of EADs after dofetilide infusion in CAVB cardiomyocytes (right panel), n = 10, 10, and 11 cells, respectively. APD_90_, action potential duration (measured at 90% repolarization); STV, short‐term variability of repolarization duration. Data shown are individual values with means ± SD, *P < .05, significantly different from baseline

Because we aimed to analyse the potential antiarrhythmic properties of LUF7244 in the CAVB dog model, we first analysed the effects of LUF7244 on adult native canine APD (Figure 4). LUF7244 was applied at 0.5, 1, 3, and 10 μM in isolated ventricular CMCs from SR and CAVB dogs. It showed dose‐dependent reduction in both APD and STV. In the EAD suppression experiment with the SR hearts, we used dofetilide‐inducible cells continuously perfused with dofetilide and LUF7244; 10‐μM LUF7244 successfully suppressed EADs in all seven cells (Figure 4b, right panel). In the EAD suppression experiment with the CAVB hearts, LUF7244 reduced the incidence of EADs to 8/10 and 9/11 at 3 and 10 μM, respectively (Figure 4c, right panel).

### 3.4 LUF7244 increases I_K_ in isolated canine CMCs and has no effect on I_Ca‐L_

**Figure 5:**
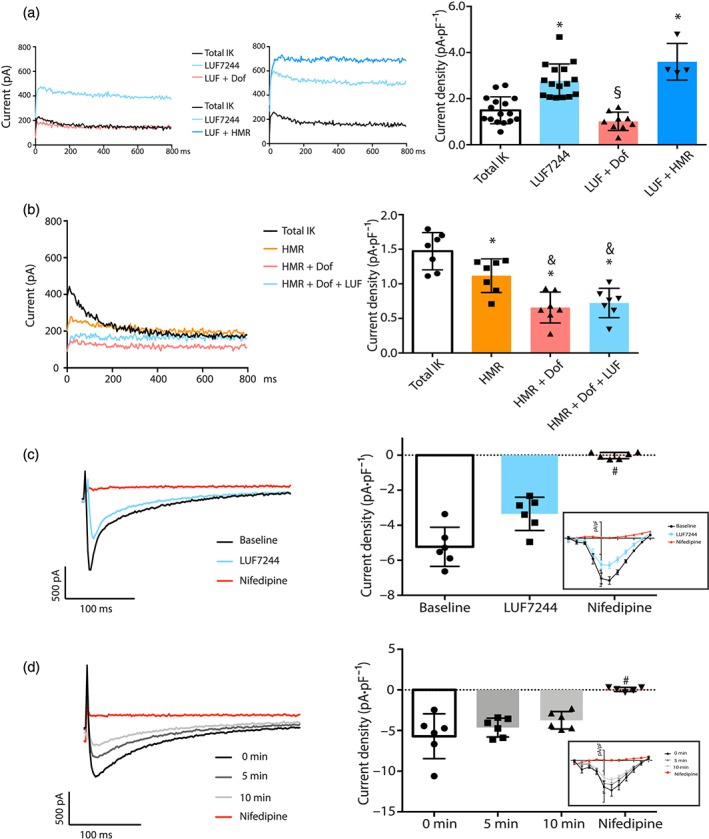
LUF7244 increases dofetilide‐sensitive I_K_ in isolated cardiomyocytes and has no effect on I_Ca‐L_. (a) Representative I_K_ traces (left panel) measured from canine cardiomyocytes. Summarized I_K_ current density at +60 mV is shown in right panel (n = 7). (b) Representative I_K_ traces (left panel) measured from canine cardiomyocytes. HMR1556 (500 nM) inhibited the I_Ks_ component of total I_K_. The total I_K_ was further blocked after 1‐μM dofetilide was applied. Summarized I_K_ current density at +60 mV after the different treatments is shown in the right panel (n = 7 cells). (c) I_Ca‐L_ measured under baseline conditions or in the presence of 10‐μM LUF7244 or 5‐μM nifedipine (n = 6). (d) Rundown measurement of I_Ca‐L_ after 0 min, 5 min or 10 min (n = 6). Current inhibition by 5‐μM nifedipine at the end of the rundown experiment. Left panel: representative traces. Right panel: peak I_Ca‐L_ density distribution at +10 mV and corresponding I‐V curves (in box) of I_Ca‐L_. Data shown are individual values with means ± SEM. *P < .05, significantly different from total I_K_, ^§^ P < .05, significantly different from LUF7244, ^&^ P < .05, significantly different from HMR, ^#^ P < .05, significantly different from baseline

We performed both prevention and suppression experiments on CMCs to test effects of LUF7244 on a group of repolarizing currents, namely, I_K_ and its HMR1556 and dofetilide‐sensitive components (Figure 5a,b). In the first experiment, LUF7244 (10 μM) was administered for 5‐min after baseline recording. Next, either 1‐μM dofetilide (I_Kr_ blocker) or 500‐nM HMR1556 (I_Ks_ blocker) combined with LUF7244 was perfused. The basal I_K_ current was approximately doubled after LUF7244, and this increase was totally inhibited by dofetilide, but not by HMR1556, indicating that the LUF7244 induced I_K_ increase was more likely to result from I_Kr_ (dofetilide sensitive) and not I_Ks_ (HMR1556 sensitive). In a second experimental set‐up, I_K_ tail current was first inhibited by dofetilide and HMR1556 to determine the contribution of I_Kr_ and I_Ks_ to total I_K_. Then 10‐μM LUF7244 was perfused and did not significantly increase I_K_ (dofetilide + HMR1556 compared with dofetilide + HMR1556 + LUF7244; Figure 5b). Collectively, these findings indicate that LUF7244 increases I_Kr_ in isolated CMCs and can partly overcome dofetilide‐mediated inhibition of I_K_. Finally, we tested for potential effects of LUF7244 on I_Ca‐L_ from CMCs. After baseline recording, 5‐min LUF7244 (10 μM) was applied and followed with 5‐μM nifedipine (Figure 5c). Next, time control experiments were performed separately for 10 min to determine rundown. No significant differences in I_Ca‐L_ densities were obtained between 10‐μM LUF7244 and time matched controls, whereas remaining current was blocked by nifedipine afterwards (Figure 5d).

### 3.5 LUF7244 decreased repolarization parameters in SR dogs

**Figure 6:**
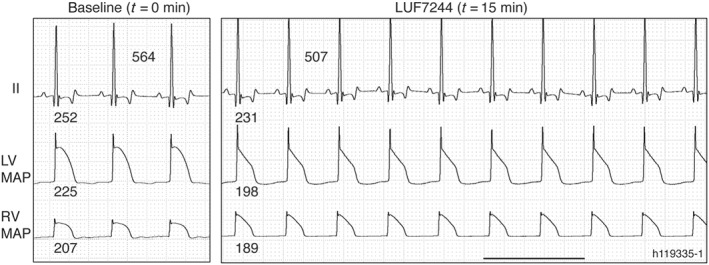
Electrophysiological effects of LUF7244 in dogs with sinus rhythm (n = 5). Representative traces of ECG measured from lead II and LV/RV MAP under baseline infusion and at the end of LUF7244 (2.5 mg·kg^−1^·15 min^−1^) infusion, respectively. Values of QT, RR, and LV/RV MAPD are presented next to the corresponding traces. Horizontal line corresponds to 1 s

In SR dogs (*n* = 5), infusion of LUF7244 (2.5 mg·kg^−1^·15 min^−1^) slightly, but not reaching significance, decreased RR duration, QTc duration, LVMAPD80, and RVMAPD80 without affecting LV STV (Figure 6, Table 1). No changes were observed in PQ and QRS interval duration or LV STV. Figure S1 displays RR and QTc data for individual animals, all showing decreases upon LUF7244 application and normalization upon washout. During infusion, LUF7244 total plasma concentration rapidly increased (0.94 ± 0.34 μM; 1.17 ± 0.50 μM at *t* = 5 and *t* = 10 min, respectively), peaked at the end of the infusion (1.75 ± 0.80 μM at *t* = 15) and then LUF7244 plasma concentrations rapidly decreased (0.27 ± 0.45 μM at *t* = 20; 0.02 ± 0.02 μM at *t* = 25).

**Table 1:** Electrophysiological parameters (in ms) in sinus rhythm dogs (n = 5) under baseline condition and after LUF7244 (15 min) administration

Parameter	Baseline	LUF7244
RR	585 ± 57	513 ± 31
PQ	130 ± 21	122 ± 22
QRS	74 ± 2	72 ± 2
QT	260 ± 20	233 ± 20
QTc^a^	296 ± 17	276 ± 17
LV_MAPD80_	200 ± 20	180 ± 19
RV_MAPD80_	183 ± 19	169 ± 17
LV_STV_	0.3 ± 0.0	0.4 ± 0.1
RV_STV_	1.0 ± 0.2	0.4 ± 0.0

*Note*. Data are expressed as mean ± *SD*.

a Van de Water QTc = QT − 0.087 * (RR − 1,000).

### 3.6 LUF7244 displayed antiarrhythmic effects against dofetilide‐induced TdP arrhythmias in CAVB dogs

Antiarrhythmic properties of LUF7244 in vivo were sequentially tested; arrhythmia inducibility by the I_Kr_ inhibitor dofetilide was tested in the first experiment, whereas in the second experiment, prevention of dofetilide‐induced arrhythmias by LUF7244 was evaluated.

#### 3.6.1 Inducibility experiment

**Figure 7:**
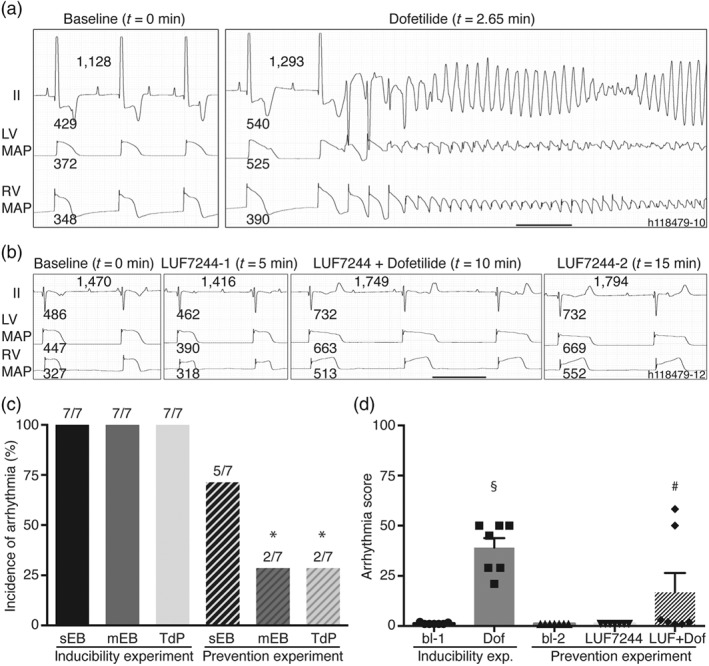
LUF7244 counteracts dofetilide‐induced TdP arrhythmia in CAVB dogs (n = 7). (a) Representative example of ECG (lead II and LV/RV MAP) under baseline infusion and at the first TdP induced by dofetilide (0.025 mg·kg^−1^·5 min^−1^). (b) The combined infusion of dofetilide and LUF7244 did not evoke TdP arrhythmia. Values of QT, RR, and LV/RV MAPD are presented next to corresponding traces. Horizontal line corresponds to 1 s. Quantification of incidence of single (sEB), multiple (mEB) ectopic beats and torsades de pointes (TdP) arrhythmia (c) and arrhythmia score (d) from both inducibility and prevention experiments. Data shown are means ± SEM, with individual values in (d). *P < .05 significantly different from TdP in inducibility experiment, ^#^ P < .05, significantly different from baseline‐2, ^###^ P < .05, significantly different from baseline‐1

In CAVB dogs (*n* = 7), dofetilide was administered as the proarrhythmia challenge (Figure 7a). As the duration of the dofetilide infusion was different in the seven inducibility experiments, due to the occurrences of TdP (see Section 2), measurements were quantified at the average infusion time of *t* = 3.9 min to determine the effects on the electrophysiological parameters in the inducibility experiments. Dofetilide infusion resulted in reproducible TdP and prolonged RR and QTc interval (Table 2) and successfully induced TdP in seven out of seven animal concomitant with an increase in arrhythmia score (1.2 ± 0.4 to 39.1 ± 12.4,). In addition, dofetilide significantly increased repolarization and arrhythmia parameters, including RV_MAPD80_ and QTc (Table 2).

**Table 2:** Electrophysiological parameters (in ms) in CAVB dogs (n = 7) under baseline condition, dofetilide, LUF7244, and LUF7244 + dofetilide administration

Parameter	Inducibility experiment		Prevention experiment		
	Baseline‐1 (0 min)	Dofetilide (3.9 min)	Baseline‐2 (0 min)	LUF7244 (5 min)	LUF7244 + dofetilide (8.9 min)
RR	1,248 ± 366	1,428 ± 393	1,485 ± 352	1,412 ± 319	1,741 ± 284
QT	429 ± 71	546 ± 69*	419 ± 64	407 ± 56	616 ± 45*
QTc^a^	401 ± 53	498 ± 44*	372 ± 58	367 ± 54	544 ± 48*
LV_MAPD80_	283 ± 33	423 ± 148	324 ± 55	329 ± 63	514 ± 79*
RV_MAPD80_	244 ± 25	334 ± 70*	272 ± 26	269 ± 32	410 ± 68*
LV_STV_	1.3 ± 1.0	3.3 ± 2.0	1.1 ± 0.6	1.1 ± 0.6	7.6 ± 6.7*
RV_STV_	2.2 ± 2.2	3.8 ± 3.8	1.0 ± 0.4	0.9 ± 0.5	5.9 ± 5.5*

*Note*. Values are represented as mean ± *SD*.

a Van de Water QTc = QT − 0.087 * (RR − 1,000).

* *P* < .05, significantly different from baseline.

#### 3.6.2 Prevention experiment

In TdP inducible CAVB dogs (*n* = 7), the combination of LUF7244 and dofetilide infusion decreased the incidence of TdP (two out of seven dogs, *P* < .05; Figure 7b,c) with a decrease in arrhythmia score (39.1 ± 12.4 to 16.8 ± 25.7) in comparison to the inducibility experiment (Figure 7d). Single ectopic beats remained present in five dogs. Electrophysiological parameters were determined at 8.9 min (5‐min LUF7244 + 3.9‐min LUF7244/dofetilide), to allow comparison with the inducibility experiment. LUF7244 did not alleviate the dofetilide‐induced increase in QTc duration and RR interval. Moreover, LV/RV_MAPD80_ and LV/RV_STV_ were significantly increased (Table 2). During the initial phase of LUF7244 infusion (0–5 min), LV/RV_STV_ was unchanged. At the end of administration (*t* = 15), LUF7244 plasma levels were 2.34 ± 1.57 μM, whereas at *t* = 30, no LUF7244 could be detected anymore. The two dogs in which TdP remained showed the lowest LUF7244 plasma concentration at *t* = 15 (0.33 and 0.85 μM). Average LUF7244 plasma levels in the five dogs free of TdP arrhythmias were 3.04 ± 1.24 μM at *t* = 15 (see Table S3 for individual values).

## 4 DISCUSSION

In the present study, we evaluated the in vivo antiarrhythmic effect of LUF7244 for the first time. We found that LUF7244 increased I_Kv11.1_ in HEK‐hERG cells and shortened APD in both hiPS‐CMCs and isolated ventricular canine cardiomyocytes. LUF7244 (10 μM) had no effects on I_KIR2.1_, I_Nav1.5_, I_Ca‐L_, and I_Ks_ but doubled I_Kr_. Moreover, LUF7244 protected 71% of the tested dogs from dofetilide‐induced TdP. In those five CAVB dogs with higher LUF7244 plasma concentration, LUF7244 protected 5/5 dogs from dofetilide‐induced TdP.

Allosteric modulators bind to a site topographically distinct from the endogenous or orthosteric site, leading to a change in receptor conformation. For a negative allosteric modulator of the K_v_11.1 channel, this will result in reduced affinity for inhibitors. Indeed, previous work had demonstrated that LUF7244 decreased the affinity of astemizole, sertindole, cisapride, or dofetilide for K_v_11.1 channels (Yu et al., 2016). While the latter was confirmed in our studies, we also found that LUF7244 was a K_v_11.1 channel activator that enhances I_Kv11.1_ even in the absence of orthosteric inhibitors. Furthermore, at a concentration above 3 μM, LUF7244 caused APD shortening in hiPS‐CMCs and isolated canine cardiomyocytes suggesting also that LUF7244 can directly act as a K_v_11.1 activator in cardiomyocytes. A number of other K_v_11.1 activators have been described, including RPR260243, (Kang et al., 2005), PD‐118057 (Zhou et al., 2005), PD‐307243 (Gordon et al., 2008), NS1643 (Hansen et al., 2006), A‐935142 (Su et al., 2009), VU0405601 (Potet et al., 2012), NS3623 (Hansen, Olesen, Rønn, & Grunnet, 2008), and ICA‐105574 (Gerlach, Stoehr, & Castle, 2009). Collectively, these show that activators can work via distinct or combined mechanisms such as slowing of deactivation, removal of inactivation, and facilitation of activation (Sanguinetti, 2014). Our data indicate that LUF7244 results in a decrease of rapid inactivation. The structurally closely related LUF7346 is thought to increase I_Kr_ by a rightward shift of voltage dependence of inactivation and a slowing of deactivation kinetics (Sala et al., 2016). Furthermore, ICA‐105574 and ML‐T531 (Zhang et al., 2012), two other structurally related activators, demonstrated type 2 activator properties (Perry et al., 2010), that is, primarily attenuating inactivation (Gerlach, Stoehr, & Castle, 2009; Zhang et al., 2012). We demonstrated earlier (Garg, Stary‐Weinzinger, & Sanguinetti, 2013) that ICA‐105574, an activator with related chemical structure, might bind at the interface between two subunits, with residue F557 being a key binding determinant. Our docking studies with LUF7244 suggest a highly similar binding mode between these two compounds. The main difference between these two compounds is the size of the molecule. Due to the larger size of LUF7244, this compound is predicted to protrude partially into the hERG cavity, where it might disturb/influence dofetilide binding. Importantly, we have recently shown that dofetilide indeed can interact not only with aromatic side chains in the cavity but also with F557 from helix S5 (Saxena et al., 2016). The amino acid sequence of the LUF7244 binding sites is completely conserved between dog and human, which is also in line with the similar effects of LUF7244 in human iPS‐derived CMCs compared to isolated canine CMCs. Whether LUF7244 binding to this site is responsible for the decrease in affinity of astemizole, sertindole, cisapride, or dofetilide remains to be elucidated.

At the cellular level, LUF7244 effectively suppressed dofetilide‐induced EADs in single ventricular dog CMCs at 3 and 10 μM, isolated either from normal (SR) or remodelled (CAVB) hearts. At 10 μM, the antiarrhythmic properties clearly go along with normalization of APD compared to baseline. In a prevention set‐up, LUF7244 (10 μM) was able to avert astemizole‐, sertindole‐, or cisapride‐induced EADs in neonatal rat ventricular CMCs (Yu et al., 2016). LUF7346 (3 μM) counteracted astemizole‐induced EADs in predisposed hiPS‐CMC bearing a proarrhythmic *KCNQ1* mutation (Sala et al., 2016).

In our prevention set‐up using CAVB dogs, LUF7244 prevented the occurrence of dofetilide‐induced TdP arrhythmias in the majority of animals (5/7), although single ectopic beats remained present in five animals. APD remained prolonged compared to baseline. This might indicate that the antiarrhythmic action of LUF7244 becomes apparent at lower concentrations than its effect on counteracting dofetilide‐induced AP prolongation, just as observed at the single cell level. Only ICA‐105574 has been tested on organ level and in vivo. ICA‐105574 prevents ventricular tachycardia/ventricular fibrillation in Langendorff‐perfused guinea pig hearts treated with moxifloxacin or chromanol 293B under hypokalaemic conditions (Meng, Shi, Li, Du, & Xu, 2013). The authors noted that ICA‐105574‐mediated restriction of drug‐induced increases in transmural dispersion of repolarization and instability of the QT interval might contribute to the antiarrhythmic mechanism (Meng, Shi, Li, Du, & Xu, 2013). In anaesthetized dogs, ICA‐105574 decreased QT(c) (20% at maximal effect) at 10 mg·kg^−1^·10 min^−1^, but not at 3 mg·kg^−1^, and increased heart rate (Asayama et al., 2013). ICA‐105574 free plasma level at maximal response was 1.7 μM. In our SR dogs, LUF7244 administration (2.5 mg·kg^−1^·15 min^−1^) was accompanied by, although not reaching significance, heart rate increase (14%) and shortened QTc (6.8%). Total plasma concentration was 1.75 μΜ at *t* = 15. We have not determined free plasma concentrations.

Antiarrhythmic properties in CAVB dogs correlate with total plasma concentration, but the source of the large variation in plasma concentrations in our seven CAVB animals is unclear. From plasma sampling in the SR animals, it is clear that T½ is extremely short and even small variations in sampling time might have large effects. In itself, a short T½ can be beneficial for a drug that is administered intravenously, as stopping the infusion terminates the effect providing full control to the interventionist.

The antiarrhythmic mechanism deployed by LUF7244 is unclear thus far. Temporal dispersion, determined as STV, in baseline is not decreased at the cell level, neither in whole animals. Also, no effect on the dofetilide‐induced increase in spatial dispersion (LV_MAPD80_‐RV_MAPD80_) was observed in CAVB animals (39 vs. 89 ms in inducibility experiment [baseline vs. dofetilide] and 52 vs. 60 vs. 104 ms in prevention experiment [baseline vs. LUF7244 vs. LUF7244 + dofetilide]). Enhanced spatial resolution in APD sampling (e.g., Bossu et al., 2018; Dunnink et al., 2016) will be necessary to determine whether local dispersion (either temporal or spatial) is targeted by LUF7244 to generate its antiarrhythmic effects.

LUF7244 may represent a novel pharmacological strategy to eliminate the unintended cardiac side effect of noncardiac drugs. Potentially, it could save numerous effective drugs. Furthermore, there are numbers of compounds that cannot pass the preclinical test because of blockade of I_Kr_. The combination of existing proarrhythmic drugs with LUF7244 would be an approach to prevent arrhythmia due to drug‐induced blockade of I_Kr_. Finally, LUF7244 and similar compounds may find a use in relieving some of the effects of long QT syndrome.

### 4.1 Limitations

The in vivo experiments were performed in CAVB dogs under anaesthesia. The anaesthetics are known to inhibit I_Kr_ current whose actions might interfere with LUF7244 action. Only a single dose of LUF7244 was tested in this study.

## AUTHOR CONTRIBUTIONS

M.Q., H.D.M.B., D.J.S., M.J.C.H., W.B.H., S.B., and D.J.B. planned and performed the experiments and analysed the data. A.W., S.H., E.C.M.L., L.H.H., A.P.IJ., M.A.V., and M.A.G.H. designed the study, planned the experiments, and analysed the data. M.Q., A.W., S.H., E.C.M.L., L.H.H., A.P.IJ., and M.A.G.H. wrote the manuscript.

## CONFLICT OF INTEREST

The authors declare no conflicts of interest.

## DECLARATION OF TRANSPARENCY AND SCIENTIFIC RIGOUR

This Declaration acknowledges that this paper adheres to the principles for transparent reporting and scientific rigour of preclinical research as stated in the *BJP* guidelines for Design & Analysis, and Animal Experimentation, and as recommended by funding agencies, publishers, and other organizations engaged with supporting research.

## Supporting information

Figure S1.
QTc (left panel) and RR (right panel) interval in individual SR dogs receiving LUF7244 (2.5 mg.kg‐1.15 min‐1, 0–15 min) and washout (15–60 min). Dog identification numbers are indicated on the right.

Table S1.
Characteristics of dogs (n = 10) involved in in vivo experiment.

Table S2.
Drug history and background information of dogs involved in isolation of CMCs (n = 44)

Table S3.
QTc interval and plasma concentration from individual dogs involved in Inducibility and Prevention experiment (n = 7)
