# Supplementary material for: LUF7244, an allosteric modulator/activator of Kv11.1 channels, counteracts dofetilide‐induced torsades de pointes arrhythmia in the chronic atrioventricular block dog model
Source: Br J Pharmacol. 2019 Aug 30;176(19):3871–85. doi: 10.1111/bph.14798 (PMC6780032; doi:10.1111/bph.14798)
Supplement: Supplementary file 1 — Figure S1. QTc (left panel) and RR (right panel) interval in individual SR dogs receiving LUF7244 (2.5 mg.kg‐1.15 min‐1, 0–15 min) and washout (15–60 min). Dog identification numbers are indicated on the right. [file BPH-176-3871-s001.pdf]

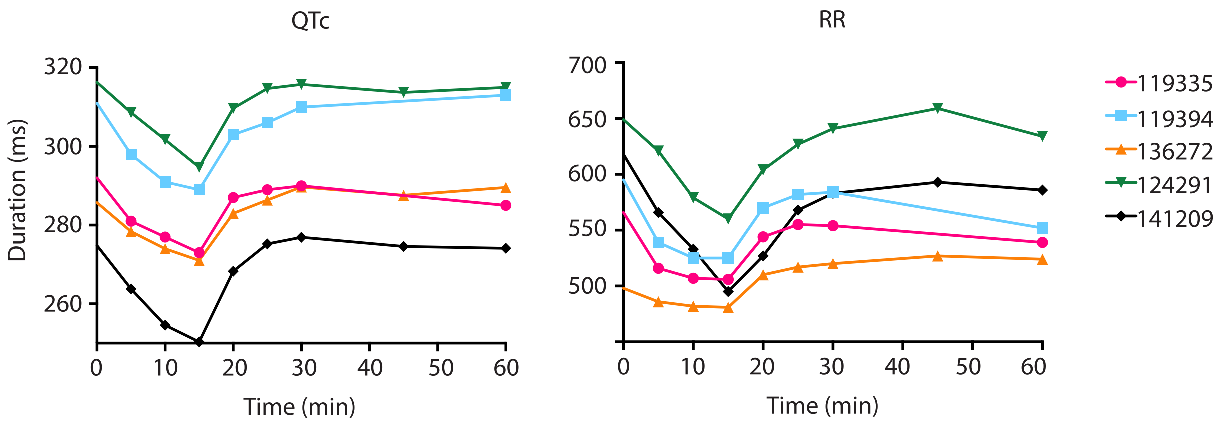


Supplemental Figure S1

QTc (left panel) and RR (right panel) interval in individual SR dogs receiving LUF7244 (2.5 mg.kg-1.15min-1, 0-15 min) and washout (15-60 min). Dog identification numbers are indicated on the right.
