# Supplementary material for: LUF7244, an allosteric modulator/activator of Kv11.1 channels, counteracts dofetilide‐induced torsades de pointes arrhythmia in the chronic atrioventricular block dog model
Source: Br J Pharmacol. 2019 Aug 30;176(19):3871–85. doi: 10.1111/bph.14798 (PMC6780032; doi:10.1111/bph.14798)
Supplement: Supplementary file 2 — Table S1. Characteristics of dogs (n = 10) involved in in vivo experiment. [file BPH-176-3871-s002.pdf]

Supplemental Table S1

Characteristics of dogs (n=10) involved in *in vivo* experiment.

Sinus Rhythm experiment				
**No.**	**Type**	**sex**	**Dog number**	**weight**
1	SR	m	119335	25 kg
2	SR	m	119394	27 kg
3	SR	f	124291	20 kg
4	SR	f	136272	19 kg
5	SR	f	141209	23 kg
**Inducibility experiment**				
**No.**	**Type[a]**	**sex**	**Dog number**	**weight**
1	CAVB2	m	119335	26 kg
2	CAVB2	m	119394	26 kg
6	CAVB2	m	118860	27 kg
7	CAVB2	m	118487	26 kg
8	CAVB2	m	118479	29 kg
9	CAVB2	m	119840	27 kg
10	CAVB2	f	133702	20 kg
**Prevention experiment**				
**No.**	**Type[a]**	**sex**	**Dog number**	**weight**
1	CAVB4	m	119335	25 kg
2	CAVB5	m	119394	26 kg
6	CAVB6	m	118860	29 kg
7	CAVB6	m	118487	27 kg
8	CAVB5	m	118479	28 kg
9	CAVB4	m	119840	27 kg
10	CAVB4	f	133702	20 kg

[a] number indicates weeks of CAVB
