# Supplementary material for: LUF7244, an allosteric modulator/activator of Kv11.1 channels, counteracts dofetilide‐induced torsades de pointes arrhythmia in the chronic atrioventricular block dog model
Source: Br J Pharmacol. 2019 Aug 30;176(19):3871–85. doi: 10.1111/bph.14798 (PMC6780032; doi:10.1111/bph.14798)
Supplement: Supplementary file 3 — Table S2. Drug history and background information of dogs involved in isolation of CMCs (n = 44) [file BPH-176-3871-s003.pdf]

Supplemental table 2. Durg history and background information of dogs invovled in CMCs isolation (n=44)

Type		N	Sex	Dog number	isolation date	Drug-1	Drug-2	Drug-3	Drug-4	Drug-5	Drugs received in isolation day[d]		interventions
Remodeled	APD	1	F	959537	19/11/2015	dofetilide 0.025mg/kg/5min (4 times)[a]	-	-	-	-	dofetilide 0.025mg/kg/5min	-	last exp, CRT7, 48 days, VVI60-CRT, multipolaire EGM LV and MAP's, non-suscept
		2	F	944840	23/11/2015	dofetilide 0.025mg/kg/5min (4 times)[a]	-	-	-	-	dofetilide 0.025mg/kg/5min	-	last exp, CRT7.5, 52 days, VVI60-CRT, multipolaire EGM LV and MAP's, suscept
		3	F	967459	30/11/2015	dofetilide 0.025mg/kg/5min (3 times)[a]	-	-	-	-	dofetilide 0.025mg/kg/5min	-	last exp, CRT8, 53 days, VVI60-CRT, multipolaire EGM LV and MAP's, suscept
		4	F	947482	02/12/2015	dofetilide 0.025mg/kg/5min (3 times)[a]	-	-	-	-	dofetilide 0.025mg/kg/5min	-	last exp, CRT8, 55 days, VVI60-CRT, multipolaire EGM LV and MAP's, non-suscept
		5	F	968447	14/01/2016	dofetilide 0.025mg/kg/5min (2 times)[a]	Hexamethonium 20mg/kg/5min(twice)[a]	-	-	-	dofetilide 0.025mg/kg/5min	Hexamethonium 20mg/kg/5min	CAVB14, last exp, test non-suscept, VVI60-RVA, RVMAP+LV EGM
		6	F	951692	27/01/2016	dofetilide 0.025mg/kg/5min (3 times)[a]	Hexamethonium 20mg/kg/5min(twice)[a]	PA-6, 0.5mg/kg/10min (once)[c]	PA-6, 2.5mg/kg/10min (once)[c]	-	dofetilide 0.025mg/kg/5min	Hexamethonium 20mg/kg/5min	CAVB15, last exp, preventie, suscept, IVR, RVMAP+LV EGM
		7	F	975257	09/02/2016	dofetilide 0.025mg/kg/5min (3 times)[a]	Hexamethonium 20mg/kg/5min(3 times)[a]	-	-	-	dofetilide 0.025mg/kg/5min	Hexamethonium 20mg/kg/5min	CAVB17, last exp, preventie, non-suscept, VVI60-RVA, RVMAP+LVEGM
		8	M	992178	20/09/2016	dofetilide 0.025mg/kg/5min (3 times)[a]	-	-	-	-	dofetilide 0.025mg/kg/5min	-	last exp, RVA8, VVI60, test non-suscept, RVMAP+LVEGM
		9	M	993506	27/09/2016	dofetilide 0.025mg/kg/5min (3 times)[a]	-	-	-	-	dofetilide 0.025mg/kg/5min	-	last exp, RVA9, VVI60, test non-suscept, RVMAP+LVEGM
		10	F	100642	03/10/2016	dofetilide 0.025mg/kg/5min (3 times)[a]	-	-	-	-	dofetilide 0.025mg/kg/5min	-	last exp, RVA9, VVI60, test non-suscept, RVMAP+LVEGM
		11	M	118479	06/06/2017	dofetilide 0.025mg/kg/5min (4 times)[a]	istaroxime, 3µg/kg/min for 1h (once)	istaroxime, 90µg/kg/5min (once)[c]	LUF7244, 2.5mg/kg/15min (once)	flunarizine 1mg/kg/1min (twice)[c]	dofetilide 0.025mg/kg/5min	-	last exp, CAVB7, denervation, RVMAP+LVEGM, NI, IVR
		12	M	118487	08/06/2017	dofetilide 0.025mg/kg/5min (5 times)[a]	istaroxime, 3µg/kg/min for 1h (once)	istaroxime, 90µg/kg/5min (once)	LUF7244, 2.5mg/kg/30min (once)	LUF7244 2.5mg/kg/15min (once)	dofetilide 0.025mg/kg/5min	-	last exp, CAVB9, denervation, RVMAP+LVEGM, NI, IVR
		13	M	119840	05/09/2017	dofetilide 0.025mg/kg/5min (4 times)[a]	sertindole 1mg/kg/5min (twice)[c]	sertindole 1mg/kg/10min (once)[c]	LUF7244 2.5mg/kg/15min (twice)[c]	-	dofetilide 0.025mg/kg/5min	-	last exp, CAVB12, LV+RV EGM, VVI45-RVA-test non-suscept
		14	F	133002	23/01/2018	dofetilide 0.025mg/kg/5min (4 times)[a]	omipalisib 7 days, 2 mg/kg/day[b]	-	-	-	dofetilide 0.025mg/kg/5min	-	last exp, ECHO-CRT8-DDD, test non-suscept, RVMAP+LVEGM, VVI60-CRT
		15	F	134466	30/01/2018	dofetilide 0.025mg/kg/5min (3 times)[a]	omipalisib 7 days, 2 mg/kg/day[b]	-	-	-	dofetilide 0.025mg/kg/5min	-	last exp, ECHO-CRT9-DDD, test non-suscept, RVMAP+LVEGM, VVI60-CRT
		16	F	135659	15/05/2018	dofetilide 0.025mg/kg/5min (2 times)[a]	ouabain 35µg/kg/min (once)	Ach 1,5-6µg/kg/min (once)[c]	isoprenaline 3-4µg/min (once)[c]	-	Ach 1,5-6µg/kg/min	isoprenaline 3-4µg/min	last exp, CAVB9, ECHO, atrial pacing, VVI40-RVA, RA+LVMAP
		17	F	139735	19/06/2018	dofetilide 0.025mg/kg/5min (2 times)[a]	ouabain 45µg/kg/min (once)	Ach 1,5-6µg/kg/min (twice)	isoprenaline 3µg/min (3 times)	-	isoprenaline 3µg/min	-	CAVB13, ECHO, IVR, aterial pacing and nervus vagus pacing
	$I_{Ca,L}$	18	M	168697	15/04/2019	dofetilide 0.025mg/kg/5min (2 times)[a]	-	-	-	-	dofetilide 0.025mg/kg/5min	-	last exp, CRT3, ECHO, test suscept, VVI60-CRT, LVEGM+RVMAP
		19	M	167917	29/04/2019	dofetilide 0.025mg/kg/5min (2 times)[a]	-	-	-	-	dofetilide 0.025mg/kg/5min	-	last exp, CRT3, ECHO, test non-suscept, VVI60-CRT, LVEGM+RVMAP
		20	M	168442	14/05/2019	dofetilide 0.025mg/kg/5min (3 times)[a]	-	-	-	-	dofetilide 0.025mg/kg/5min	-	last exp, RVA4, ECHO, test non-suscept, VVI60-RVA, LVEGM+RVMAP
		21	F	168361	21/05/2019	dofetilide 0.025mg/kg/5min (3 times)[a]	-	-	-	-	dofetilide 0.025mg/kg/5min	-	last exp, RVA4, ECHO, test non-suscept, VVI60-RVA, LVEGM+RVMAP
		22	F	168646	28/05/2019	dofetilide 0.025mg/kg/5min (3 times)[a]	-	-	-	-	dofetilide 0.025mg/kg/5min	-	laste exp, RVA5, ECHO, test non-suscept, VVI60-RVA, LVEGM+RVMAP
Sinus Rhythm	APD	23	M	-	16/02/2016								WilResearch Pharmacokinetics
		24	M	-	23/02/2016								WilResearch Pharmacokinetics
		25	M	-	01/03/2016			no information			no information		WilResearch Pharmacokinetics
		26	M	-	08/03/2016								WilResearch Pharmacokinetics
		27	F	-	15/03/2016								WilResearch Pharmacokinetics
		28	F	-	22/03/2016								WilResearch Pharmacokinetics
		29	M	Beagle	23/05/2016	-	-	-	-	-	-	-	Education only
		30	M	Beagle	25/05/2016	-	-	-	-	-	-	-	Education only
		31	M	-	12/07/2016								WilResearch Pharmacokinetics
		32	M	-	19/07/2016								WilResearch Pharmacokinetics
		33	M	-	25/07/2016								WilResearch Pharmacokinetics
		34	M	-	02/08/2016								WilResearch Pharmacokinetics
		35	M	-	09/08/2016			no information			no information		WilResearch Pharmacokinetics
		36	F	-	19/12/2017								Charles River Pharmacokinetics
		37	F	-	16/01/2018								Charles River Pharmacokinetics
		38	F	-	06/03/2018								Charles River Pharmacokinetics
		39	F	-	13/03/2018								Charles River Pharmacokinetics
		40	F	187825	24/07/2018	-	-	-	-	-	-	-	knee operation
	IK	41	M	167925	12/03/2019	dofetilide 0.025mg/kg/5min (2 times)[a]	omipalisib 7 days, 2 mg/kg/day[e]	-	-	-	dofetilide 0.025mg/kg/5min	-	last exp, day 15 VDD, test non-susceptibility, VVI60-RVA, RV-LVMAP, day 7 OMI
		42	M	167942	19/03/2019	dofetilide 0.025mg/kg/5min (2 times)[a]	omipalisib 7 days, 2 mg/kg/day[e]	-	-	-	dofetilide 0.025mg/kg/5min	-	last exp, day 14 VDD, test non-susceptibility, VVI60-RVA, RV-LVMAP, day 7 OMI
		43	M	167895	26/03/2019	dofetilide 0.025mg/kg/5min (2 times)[a]	omipalisib 7 days, 2 mg/kg/day[e]	-	-	-	dofetilide 0.025mg/kg/5min	-	last exp, day 15 VDD, test non-susceptibility, VVI60-RVA, RV-LVMAP, day 7 OMI
		44	M	168221	03/04/2019	dofetilide 0.025mg/kg/5min (2 times)[a]	omipalisib 7 days, 2 mg/kg/day[e]	-	-	-	dofetilide 0.025mg/kg/5min	-	last exp, day 16 VDD, test non-susceptibility, VVI60-RVA, RV-LVMAP, day 7 OMI
