# Supplementary material for: LUF7244, an allosteric modulator/activator of Kv11.1 channels, counteracts dofetilide‐induced torsades de pointes arrhythmia in the chronic atrioventricular block dog model
Source: Br J Pharmacol. 2019 Aug 30;176(19):3871–85. doi: 10.1111/bph.14798 (PMC6780032; doi:10.1111/bph.14798)
Supplement: Supplementary file 4 — Table S3. QTc interval and plasma concentration from individual dogs involved in Inducibility and Prevention experiment (n = 7) [file BPH-176-3871-s004.pdf]

Supplemental Table S3
QTc interval and plasma concentration from individual dogs involved in Inducibility and Prevention experiment (n=7)

	Dog number	Inducibility experiment		Prevention experiment						
				QTc (ms)[c]			Plasma concentration (ng/mL)			AS
		Baseline-QTc (0 min)	Dofetilide-QTc (3.9 min)	Baseline (0 min)	LUF7244 (5 min)	LUF7244 +Dofetilide (8.9 min)	Baseline (0 min)	LUF7244 (15 min)	washout (30 min)	
Inducible	**119335**	334±2	456±11	336±4	328±7	567±3	10	1133	0	1
	**118487**	333±1	543±13	325±2	325±2	566±8	0	1353	0	3
	**118860**[b]	471±2	616±4	449±3	435±3	665±7	0	693	0	2
	**118479**	438±3	444±8	455±9	444±3	608±2	0	654	0	2
	**133702**	433±6	524±8	319±8	315±6	467±16	0	1734	17	1
N-I[a]	**119394**	420±4	480±43	363±2	372±5	536±7	0	312	0	50
	**119840**	445±5	540±6	433±2	416±4	520±18	0	120	0	58.3

[a] Non-Inducible dog
[b] dog 118860 was paced at VVI60, so only QT is shown
[c] Van de Water QTc = QT-0.087(RR-1000)
values are represented as mean ± SD. AS indicates arrhythmia score
